# Excision repair cross-complementing group-1 (ERCC1) induction kinetics and polymorphism are markers of inferior outcome in patients with colorectal cancer treated with oxaliplatin

**DOI:** 10.18632/oncotarget.27140

**Published:** 2019-09-17

**Authors:** Devika Rao, Atrayee Basu Mallick, Titto Augustine, Cecilia Daroqui, Jeeshan Jiffry, Amartej Merla, Imran Chaudhary, Raviraja Seetharam, Arjun Sood, Srikanth Gajavelli, Santiago Aparo, Lakshmi Rajdev, Andreas Kaubisch, Jennifer Chuy, Abdissa Negassa, John M. Mariadason, Radhashree Maitra, Sanjay Goel

**Affiliations:** ^1^Department of Medical Oncology, Montefiore Medical Center, Bronx, New York, USA; ^2^Department of Medicine, Albert Einstein College of Medicine, Bronx, New York, USA; ^3^Department of Epidemiology and Biostatistics, Albert Einstein College of Medicine, Bronx, New York, USA; ^4^Gastrointestinal Cancers Program and Oncogenic Transcription Laboratory, Olivia Newton-John Cancer Research Institute, La Trobe University School of Cancer Medicine, Melbourne, Australia

**Keywords:** oxaliplatin, colorectal cancer, ERCC1, FOLFOX, resistance

## Abstract

**Background:**

ERCC1, a component of nucleotide excision repair pathway, is known to repair DNA breaks induced by platinum drugs. We sought to ascertain if ERCC1 expression dynamics and a single nucleotide polymorphism (SNP) rs11615 are biomarkers of sensitivity to oxaliplatin therapy in patients with colorectal cancer (CRC).

**Methods:**

Western blot and qPCR for ERCC1 expression was performed from PBMCs isolated from patients receiving oxaliplatin-based therapy at specified timepoints. DNA was also isolated from 59 biorepository specimens for SNP analysis. Clinical benefit was determined using progression free survival (PFS) for metastatic CRC.

**Results:**

ERCC1 was induced in PBMC in response to oxaliplatin in 13/25 patients with mCRC (52%). Median PFS with ERCC1 induction was 190d compared to 237d in non-induced patients (HR 2.35, CI 1.005-5.479; p=0.0182). *ERCC1* rs11615 SNP analysis revealed that 43.3% harbored C/C, 41.2%-T/C and 15.5%-T/T genotype. Median PFS was significantly lower with C/C or T/C (211 and 196d) compared to T/T (590d; p=0.0310).

**Conclusions:**

ERCC1 was induced in a sub-population of patients undergoing oxaliplatin treatment, which was associated with poorer outcome, suggesting this could serve as a marker of oxaliplatin response. C/C or C/T genotype in *ERCC1* rs11615 locus decreased benefit from oxaliplatin.

## INTRODUCTION

The platinum group of cancer chemotherapeutic agents includes cisplatin, its analog carboplatin, and oxaliplatin, however, only oxaliplatin is effective in the treatment of colorectal cancer (CRC). Oxaliplatin (trans-L-1,2-diamino cyclohexane oxalatoplatinum) is a third-generation platinum compound that is highly effective when used in combination with 5-FU and is a standard treatment option for lymph node positive colon cancer, and as frontline therapy in the advanced setting [1–4]. CRC is the second-leading cause of cancer-related deaths in the US among both men and women, with an estimated mortality of 51,020 in 2019 [5]. Patients who develop metastatic disease (mCRC) have a relatively poor outcome, with median survival of 24-30 months and 5-year survival rates of ~14% [6]. In addition to CRC, the platinum drugs are an important class of therapeutic agents for a wide variety of other cancers, including lung, breast, esophageal, gastric, ovarian, testicular, cervical, endometrial, and bladder cancer; however, their efficacy is limited by both inherent and acquired drug resistance [7].

Tumor cell resistance to oxaliplatin appears to be multifactorial, with the nucleotide excision repair (NER) pathway playing a major role [8]. NER is carried out by a multienzyme complex and involves a stepwise process of DNA damage recognition, incision, excision, repair, synthesis, and ligation [9, 10]. Excision repair cross complementing group 1 (ERCC1), along with xeroderma pigmentosa (XPA), form a critical heterodimer, active in the NER pathway, cleaving DNA upstream of the site of DNA damage [9, 11–18]. There is evidence that the relative level of *ERCC1* mRNA is a good marker for NER activity in human cancer cells, but it is unclear whether expression of this gene is important for other pathways of DNA repair [19, 20]. It has been previously shown that a high basal levels of ERCC1 is associated with poorer survival in patients with mCRC treated with oxaliplatin [21], although, surprisingly, no difference in tumor response was found [22, 23]. Although platinum drugs predominantly result in bulky DNA-distorting adducts and elicit NER, they can also induce inter-strand crosslinks that are repaired by the Fanconi pathways during S phase. Furthermore, other cellular repair mechanisms, such as recombination or mismatch repair, can affect antitumor efficiency of platinum compounds. Therefore, ERCC1 definitely has some limitations as a biomarker in completely evaluating all relevant pathways involved in repair of platinum-induced DNA damage [24]. As a result, while there have been promising data supporting ERCC1 expression levels as biomarkers in pre-clinical and small clinical models, when expanded to large randomized controlled trials (RCT), the scientific community has been unable to establish the utility of ERCC1 as a predictive biomarker.

In addition to its potential as a basal biomarker of oxaliplatin response, studies in colorectal and ovarian cancer cells have demonstrated that ERCC1 expression is induced upon treatment with platinum-based agents [25], while work from our group has demonstrated that the extent of ERCC1 induction in CRC cell lines treated with oxaliplatin, can distinguish sensitive from resistant cell lines [26]. Consistent with a direct role for ERCC1 in determining oxaliplatin response, silencing of ERCC1 mRNA using small interfering RNAs (siRNA) was able to render a formerly resistant cell line sensitive to oxaliplatin. In this study we sought to extend this finding by examining whether oxaliplatin can also induce ERCC1 gene expression in peripheral blood mononuclear cells (PBMC) and whether this could serve as a surrogate marker of oxaliplatin response in patients with CRC. Measurements on tumor biopsies are challenging due to inherently low adduct levels and difficulties in obtaining biopsies at multiple time points, when the resulting data would be most informative. Several reports have documented associations between drug–DNA adduct levels in surrogate tissues and clinical response and toxicity [27–32]. The platinum–DNA adduct formation in PBMCs was found to more predictive for tumor response to platinum-based therapy than previous platinum-based therapy, stage of disease, histological type and tumor grading. Therefore, it is reasonable to hypothesize that changes in expression of ERCC1 levels in PMBC can be used as a surrogate to tumor tissue.

Additionally, we assessed the prevalence of a single nucleotide polymorphism (SNPs) in *ERCC1* and its relation to patient outcomes. Several common and functional SNPs of *ERCC1* have been identified, of which *ERCC1* rs11615 (C118T) is considered to have some effects on *ERCC1* mRNA expression [33]. However, published reports of an association between NER SNPs and clinical outcome of platinum-based chemotherapy from individual studies are not consistent. A large meta-analysis of 17 published studies, including 1787 patients, attempted to focus on this issue [34]. However, among the 17 studies included, 8 originated from eastern Asia, 7 from Europe/Australia and only 2 from USA. Hence the true distribution of NER SNPs in the American population is not well established.

## RESULTS

### Patient characteristics

**Figure 1:**
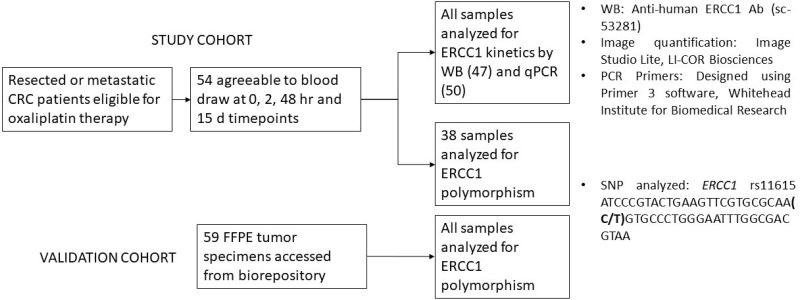
A schematic representation of the study workflow. 54 patients were consented for blood draw to assess ERCC1 kinetics. Expression levels in all patients were assessed by either Western blot or polymerase chain reaction. 43/54 patients had analysis by both modalities. 38/54 samples were additionally assessed for presence of *ERCC1* rs11615 SNP. This was based on availability of residual sample. For validation of *ERCC1* rs11615 SNP analysis 59 individual samples were additionally obtained from an institutional biorepository which stores excised tumors as Formalin Fixed Paraffin Embedded (FFPE) tissue blocks.

Figure 1 depicts the workflow of the study. Fifty-four patients consented to the study and underwent serial blood sampling. Blood samples were collected from every patient for at least at two of the four prespecified time points. There were six patients who did not have sampling at all time points, 3 of whom were from the mCRC cohort. Baseline demographic characteristics are listed in Table 1. Of the 54 patients, 25 had mCRC, of whom one was excluded from analysis due to mortality prior to completing one cycle of therapy. Twenty nine patients had limited stage disease - 3 with stage II and 26 with stage III CRC. Among the 54 patients, 4 received capecitabine along with oxaliplatin (XELOX) including 1 patient with mCRC, and the 50 received infusion 5-fluorouracil in combination with oxaliplatin (FOLFOX). All 24 patients in the mCRC cohort experienced progression of disease while on oxaliplatin and hence no patients were censored.

**Table 1:** Baseline demographic and clinical characteristics of patients consented for blood draw

	Limited stage disease N=29 (%)	Metastatic disease N=25 (%)
Gender -Male -Female	13 (45) 16 (55)	13 (52) 12 (48)
Median age (yrs.)	59	59
Race -White -Black -Hispanic -Asian -Unknown	6 (21) 6 (21) 15 (52) 1 (3) 1 (3)	4 (16) 8 (32) 11 (44) 1 (4) 1 (4)
Sidedness -Right -Left	8 (28) 21 (72)	9 (36) 16 (64)
KRAS -Mutated -Wild Type -Not performed	3 (10) 6 (21) 20 (69)	10 (40) 10 (40) 5 (20)

This table lists the baseline demographic and clinical characteristics of 54 patients who consented to blood draw for analysis of ERCC1 kinetics in PBMC. The patients are divided by stage of malignancy into limited stage (clinical stage II-III) and metastatic disease (clinical stage IV). Race was determined by patient self-identification. Based on site of origin of primary tumor, patients were categorized into right (cecum, ascending colon, hepatic flexure, upto mid-transverse colon) or left (distal transverse colon, splenic flexure, descending colon, sigmoid colon and rectum) sided. KRAS mutation status was identified, and as expected was performed less frequently in limited stage disease.

### Baseline levels of *ERCC1* do not correlate with patient outcomes

**Figure 2:**
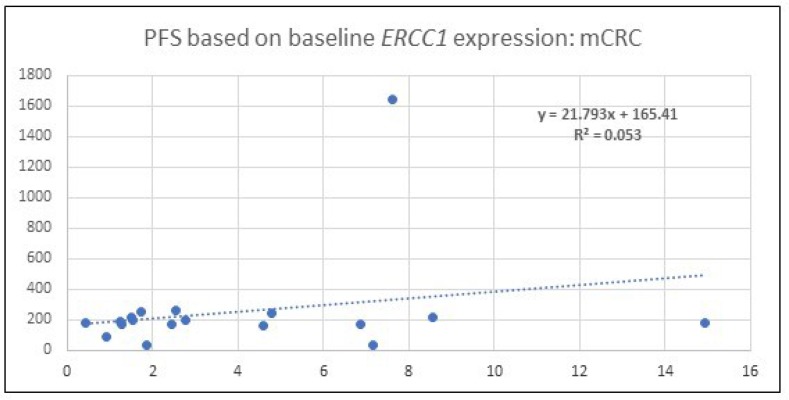
A scatter plot which depicts the PFS among patients with mCRC. Correlation is made with baseline, 0 hour, *ERCC1* expression identified by qPCR after normalization with the housekeeping gene, *GAPDH*. R^2^ value indicates no significant correlation exists.

Baseline *ERCC1* levels of all patients (50) with qPCR analysis was obtained. The baseline expression in mCRC patients when normalized to GAPDH ranged between 0.45 and 14.94. In this subset, comparison of level with PFS was performed and is depicted in Figure 2. Given the low R^2^ it is evident that there is poor correlation between baseline *ERCC1* levels and PFS.

### Oxaliplatin induces *ERCC1* mRNA expression in PBMCs

**Figure 3:**
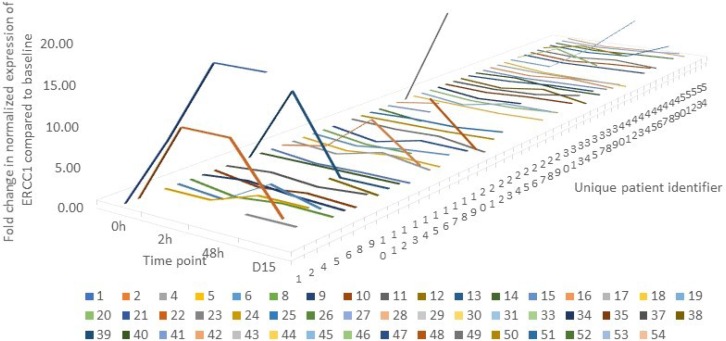
A line graph depicting the pattern of change in ERCC1 expression over time in each individual patient (n=50) where mRNA expression was quantified by qPCR. The graph is censored at 20 to allow for graphical representation. The lowest expression was seen in patient 21 at 2 hours where ERCC1 level was suppressed to 2% of baseline levels. Contrarily, the highest expression was in patient 1 at 48 hours, where *ERCC1* level was 400% of baseline.

To determine if oxaliplatin induces *ERCC1* mRNA expression in PBMCs, we performed qPCR on RNA isolated from PBMCs pre- and pos-treatment, at 4 time points mentioned under methods. Samples for mRNA expression were available for 50 of the 54 patients who participated in the study. The pattern of fluctuation is depicted in Figure 3. The four patients who did not have qPCR readings are excluded from the graph. The lowest expression of *ERCC1* was seen in patient 21 at 2 hours where ERCC1 level was suppressed to 2% of baseline levels. Contrarily, the highest expression was in patient 1 at 48 hours, where *ERCC1* level was 400% of baseline.

Of the 50 patients, 25 (50%) demonstrated an increase in *ERCC1* expression post-oxaliplatin treatment, 14 patients (28%) had a decrease in expression, and 11 (22%) did not present any change in expression. This initial result suggests that *ERCC1* is a highly dynamic gene whose expression can be altered (increased or decreased) in PBMCs upon exposure to oxaliplatin.

### Oxaliplatin treatment induces ERCC-1 protein expression in PBMCs

**Figure 4:**
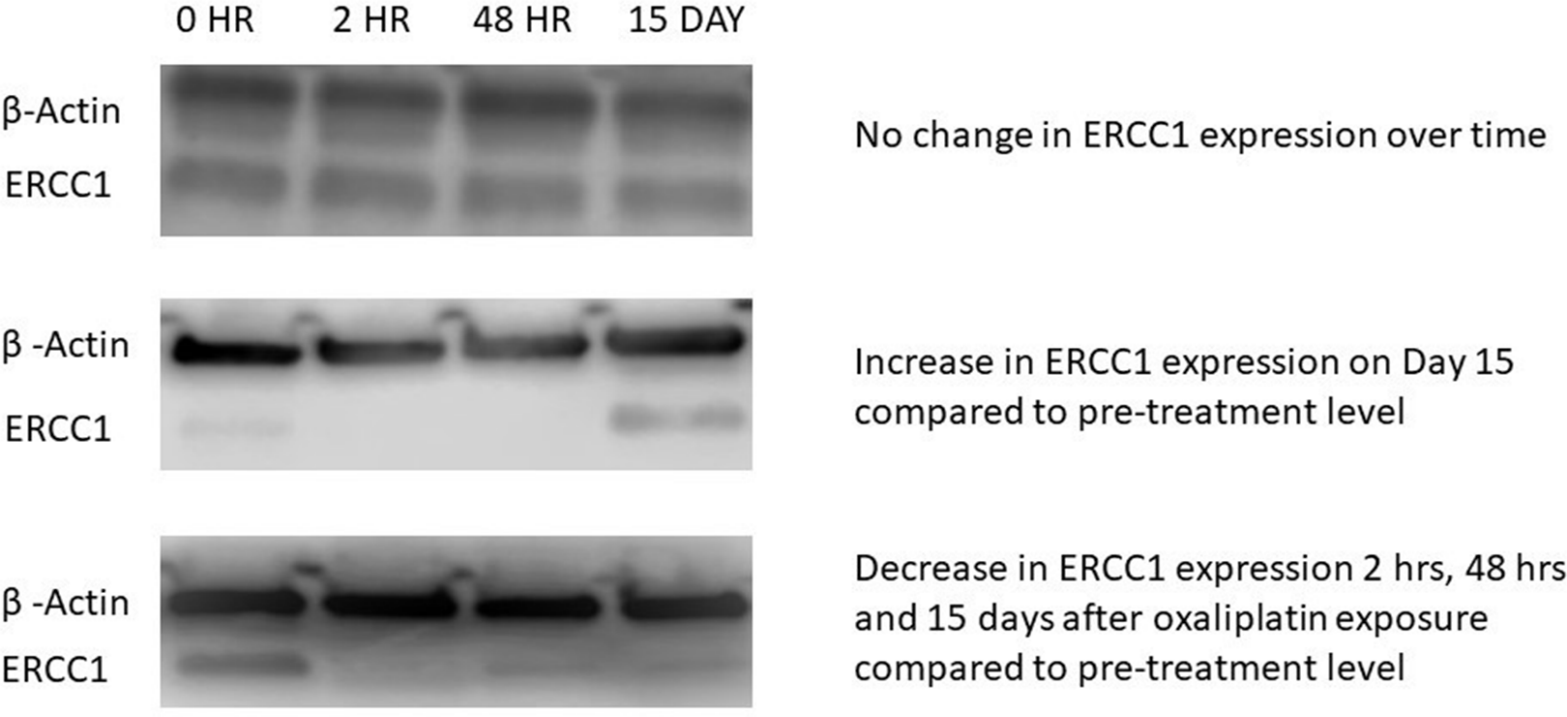
Western blot (WB) images from three representative patients. The superior band indicates expression of a housekeeping gene, β -actin, which served as internal control. The inferior band represents the gene of interest, ERCC1. Each patient had expression analyzed at four time points: 0, 2, 48 Hour and 15 Day. The first patient did not show change in ERCC1 expression over time, assessed by band intensity remaining equal over time. Patient 2 had an increase in ERCC1 band intensity at the 15 Day time point compared to 0 Hour, while Patient 3 showed the maximum ERCC1 expression at 0 Hour. In this case, there was a decrease in expression over the other three time points.

We next determined if oxaliplatin induces ERCC1 protein expression in PBMCs, by performing Western blot analysis on proteins isolated from PBMCs pre and post oxaliplatin treatment. WB analysis was performed on blood samples from 47 patients, drawn at two or more time points of oxaliplatin treatment. Of the 47 evaluable patients, 26 (55.3%) demonstrated an increase in ERCC1 protein expression, 9 (19.1%) showed a decrease in expression, and 12 (25.5%) experienced no change in expression (Figure 4).

### Correlation between protein and mRNA expression dynamics

We next performed a correlation analysis to examine the association between ERCC1 mRNA and protein induction following oxaliplatin treatment. Of the 50 patients whose samples were analyzed by qRT-PCR, corresponding Western blot data was obtained for 43 patients. Of these, induction of *ERCC1* mRNA and protein were concordant in 23 patients (53.5%), indicating a discrepancy between *ERCC1* mRNA and protein induction. For purpose of analysis, discordant patients were allotted a final “increase” status based on the PCR results if there was at least a 50% increase in *ERCC1* expression between pre- and post-treatment samples.

### ERCC1 induction is associated with reduced benefit from oxaliplatin treatment in patients with metastatic CRC

**Figure 5:**
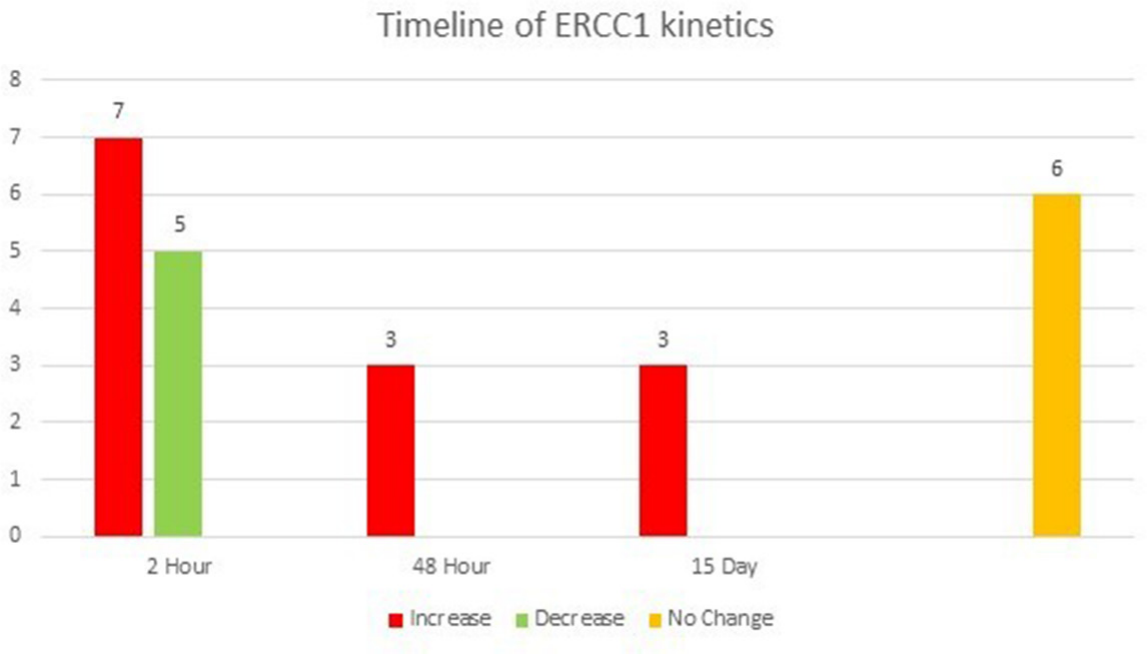
A graphical representation of the ERCC1 kinetics in patients with mCRC over time. 13 patients had an increase in ERCC1 expression, with early increase, at the 2 hour mark, being seen in 7/13 patients. 5 patients had a decrease in ERCC1 expression, with all showing the change as early as the 2-hour time point. Eighteen patients with mCRC had a change in ERCC expression, of whom 12 (67%) demonstrated this at 2 hours. Six patients had no change in expression at any time point.

**Figure 6:**
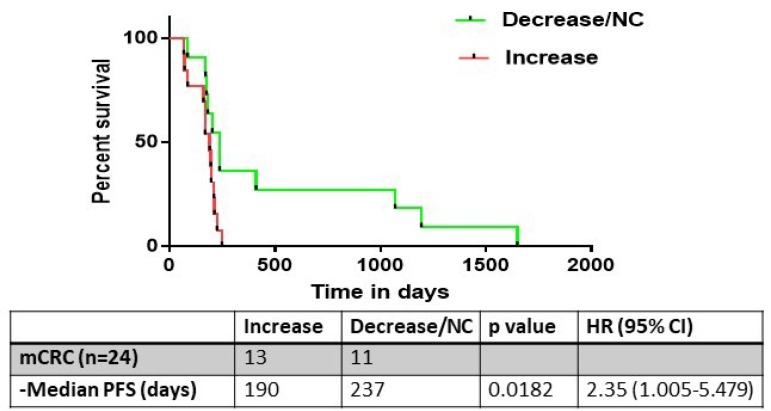
The progression free survival (PFS) analysis of metastatic colorectal cancer patients who underwent evaluation of ERCC1 kinetics (n=24). 13/24 had an increase in ERCC1 expression, with a corresponding median PFS of 190 days. This was significantly lower than the PFS of patients who had no change or decrease in ERCC1 expression kinetics (237 days, p= 0.018).

We next performed a subset analysis on the 24 patients in the cohort who had mCRC. Of these patients, a final increase in *ERCC1* expression compared to baseline was observed in 13 patients (52%), while no change or a decrease in *ERCC1* expression was observed in 11 patients (48%) (Figure 5). Notably, median PFS was significantly lower in the patients in which *ERCC1* expression was induced post-treatment (190 vs 237 days, log-rank test HR 2.35, CI 1.005-5.479; p = 0.0182) (Figure 6).

Comparatively, 19 out of the 29 patients (65.5%) with limited stage disease had an induction of the *ERCC1* expression, while in 10 patients (34.5%), *ERCC1* expression did not change or decreased. However, in these patients change in expression did not correlate with relapse free survival (RFS).

### *ERCC1* SNP distribution among races and influence on patient outcome

### Study cohort

The *ERCC1* gene contains a C/T polymorphism (rs11615) in its coding sequence. While both variants encode the same amino acid, asparagine (synonymous SNP), the C>T transition results in the conversion of a commonly used codon (AAC) into an infrequently used codon (AAT) which may have implications for *ERCC1* expression. This SNP was assessed in 38/54 patient samples, of whom 19 (50%) had stage IV disease. On correlation of genotype with survival, median PFS on oxaliplatin was significantly lower in patients harboring the C/C or heterozygous genotypes (79 and 213 days respectively) compared to patients harboring the homozygous T/T genotype (640 days; log-rank test p=0.0139).

**Figure 7:**
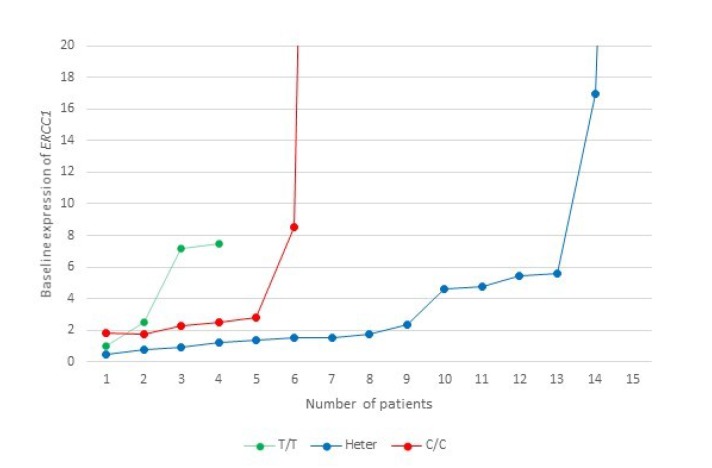
A representation of the baseline ERCC1 mRNA expression levels level in relation to ERCC1 rs11615 SNP in the study cohort. This graph is censored at 20, while the highest expression in the C/C group is 126 (n=1) and in the heterozygote group is 77 (n=1).

On correlation of baseline mRNA expression with SNPs, patients with C/C genotype had the highest expression (mean, 20.86), followed by heterozygotes (mean, 8.46) and T/T homozygotes (mean, 4.54) (Figure 7). This finding, however, did not achieve statistical significance (ANOVA, F=0.589, p=0.563).

### Validation Cohort

**Figure 8:**
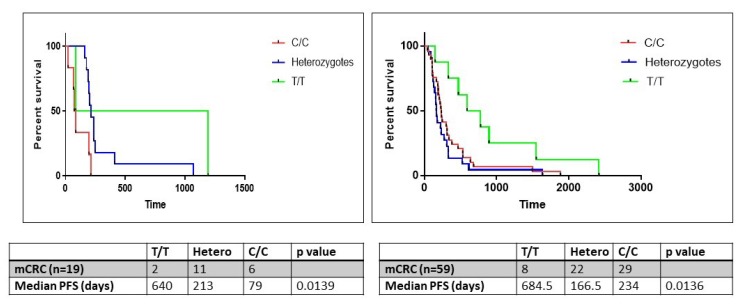
The progression free survival (PFS) analysis in the study (n=19) and validation (n=59) cohorts of patients with metastatic colorectal cancer wherein tumor tissues were obtained from an institutional biorepository. Samples were analyzed for *ERCC1* rs11615 SNP. Patients were categorized into homozygous (C/C, T/T) and heterozygous (T/C) genotypes.

In order to further validate the influence of SNP on PFS while on oxaliplatin, DNA was obtained from FFPE tissues of 59 patients with metastatic colorectal cancer, stored in an institutional biorepository. Basic demographic information of this validation cohort is presented in Table 2. In this cohort as well, median PFS on oxaliplatin was significantly lower in patients harboring the C/C or heterozygous genotypes (234 and 166.5 days respectively) compared to patients harboring the homozygous T/T genotype (684.5 days; log-rank test p=0.0136) (Figure 8).

**Table 2:** Baseline demographics of patients in the validation cohort for SNP analysis

	mCRC n=59 (%)
Gender -Male -Female	16 (27) 43 (73)
Median age (yrs.)	58
Race -White -Black -Hispanic -Asian -Unknown	10 (17) 26 (44) 15 (25) 1 (2) 7 (12)

This table lists the baseline demographic and clinical characteristics of the 59 mCRC patients whose tissue samples were extracted from the institutional biorepository to serve as a validation cohort for SNP analysis. Race was determined by patient self-identification.

### Combined analysis of racial distribution

Among all the 97 samples where *ERCC1* SNP was analyzed, the homozygous C/C genotype was observed in 42 patients (43.3%), the homozygous T/T genotype was observed in 15 patients (15.5%), and 40 patients (41.2%) harbored the heterozygous genotype.

Notably, a strong association between *ERCC1* SNP genotype and racial distribution was observed, with the prevalence of the homozygous C/C genotype highest in blacks (76%), followed by Asians (33%), Hispanics (30%), persons of unknown race (25%) and whites (6%) (Fisher exact test p=0.0002) (Table 3).

**Table 3:** Patient distribution by race among the homozygous (C/C, T/T) and heterozygous (T/C) genotypes of the *ERCC1* SNP

	**T/T** **N=15**	**T/C** **N=40**	**C/C** **N=42**	**Fisher Exact test, p= 0.0002**
Black (%)	0 (0)	9 (24)	28 (76)	
White (%)	5 (31)	10 (63)	1 (6)	
Hispanic (%)	7 (21)	16 (49)	10 (30)	
Asian (%)	0 (0)	2 (67)	1 (33)	
Unknown (%)	3 (38)	3 (37)	2 (25)	

This table depicts the distribution of *ERCC1* rs11615 SNP between the homozygous (C/C, T/T) and heterozygous (T/C) genotypes. Patients are further classified by race. On analyzing this distribution with a Fisher exact test, statistical significance was observed with p=0.00023.

## DISCUSSION

The outcome of patients with metastatic (stage IV) CRC has improved considerably over the past decade, from a median survival of 6 months with best supportive care, to 24-30 months with the incorporation of the newer cytotoxic drugs (including irinotecan and oxaliplatin) and the monoclonal antibodies bevacizumab, panitumumab and cetuximab [35]. While immunotherapy has revolutionized therapy options in many solid tumors, checkpoint inhibitors are only active in the subset of mCRC patients with microsatellite-unstable (MSI) disease, who represent <4% of the mCRC population [36].

Therefore, a combination of oxaliplatin with 5-FU and leucovorin continues to remain the standard frontline treatment for patients with metastatic disease, and also in adjuvant therapy for lymph node positive (stage III) CRC [3, 4]. Response rates in these patients is ~50% [37], necessitating the need to identify biomarkers which can predict the likelihood or response [38]. Additionally, analyses of the large phase III National Surgical Adjuvant Breast and Bowel Project (NSABP) C-07 study and the MOSAIC study suggest that despite being active in patients with stage III (and high-risk stage II) CRC, oxaliplatin failed to eradicate micro metastatic disease in 30% of the patients [4, 39]. Thus, inherent resistance to platinum drugs continues to be a major detrimental factor in the therapeutic outcome of cancer patients. ERCC1 is an important component of the NER pathway, and therefore may be an important determinant of response to these drugs [40].

While multiple clinical trials have suggested that a high baseline tumor expression of ERCC1 protein or mRNA [21, 22, 41] may serve as a biomarker for relative resistance to platinum compounds, no studies have examined the relationship between ERCC1 induction in response to oxaliplatin treatment and patient outcome. In this study, we demonstrate that ERCC1 expression is induced in a subset of patients with CRC. Notably, only a modest correlation was observed between mRNA and protein induction suggesting post-transcriptional or post-translational regulation may also be involved in ERCC1 expression although this requires further study. Another possibility is that the induction of gene expression is an early event and hence detected at the 2-hour sample, while protein induction is delayed, possibly occurs at an intermediary time point. This would need further study with focus on obtaining blood samples at time points between 2 and 48 hours to assess for protein changes.

Importantly, we observed that progression free survival was significantly shorter in patients who showed *ERCC1* gene induction following oxaliplatin treatment compared to those who did not, suggesting monitoring *ERCC1* induction in PBMCs may be a potential means of determining likelihood of benefit from oxaliplatin-based treatment. Our finding that *ERCC1* induction can be monitored in peripheral blood, at an early time point of 2 hours post-infusion, makes this approach especially appealing for clinical implementation.

ERCC1 has demonstrated promise as a predictive and prognostic marker in solid tumors such as non-small cell lung (NSCLC), ovarian, pancreatic and colorectal cancer [42–44]. Our observation that induction of ERCC1 expression is a predictor of oxaliplatin resistance represents a novel approach as it assesses kinetics of induction rather than baseline expression of the gene. This observation is consistent with our group’s *in vitro* data that showed similar findings in cell culture [26].

Moreover, we noted that presence of *ERCC1* rs11615 SNP independently influences the outcome in patients with mCRC, which was validated in a second cohort of patients. This synonymous polymorphism (Asn118Asn) has been associated with decreased *ERCC1* mRNA and protein levels in prior studies [45]. However, we did not find a correlation between the SNP and baseline ERCC1 levels in our analysis. It is possible that our findings are limited by the small number of samples where correlation of baseline expression with SNP status could be performed (38/97). The rs11615 SNP has been reported in the literature to be associated with drug resistance [46, 47]. A phase II study where patient stratification to chemotherapy was based on pharmacogenetic profile (*ERCC1* SNP being one among four studied genes) failed to demonstrate any benefit in terms of efficacy or as regards toxicity profile [48]. However, in this study, the C/C genotype was considered favorable and treated with oxaliplatin based therapy, which is contrary to our findings. Hence, failure of the study may be secondary to inappropriate patient allotment. Further, in studies performed across various solid tumors, persons with the T/T genotype appear to derive the most benefit from platinum-based chemotherapy, while the C/C group had the worst outcome [49–52].

Anti-cancer therapy in mCRC is in dire need of biomarkers that can guide the clinician in selecting the right drug for the right patient. The use of *KRAS* mutation as a biomarker of exclusion for patients receiving anti-EGFR therapy, specifically cetuximab [53] and panitumumab [54] was a significant landmark in the advancement of cancer therapy. However, much work needs to be done to make the approach to personalized medicine a true reality. Our findings are encouraging in that they identify a method for rapidly determining likelihood of benefit to oxaliplatin by measuring ERCC1 induction in a convenient and repeatedly accessible tissue (PBMC). To accomplish this goal, further study and confirmation of our findings are required. Furthermore, our findings represent one of many steps required to help patients by providing them better outcomes and simultaneously minimizing toxicity of chemotherapy drugs by limiting the use of drugs less effective in a subset of patients.

## PATIENTS AND METHODS

### Patient selection

Patients with either surgically resected colon or rectal cancer or with unresectable metastatic disease who were candidates for oxaliplatin-based chemotherapy (with either 5-FU or capecitabine, with or without bevacizumab) were included in the study. Eligible patients were adults (age ≥ 18 years), with an ECOG performance status of 0-2. Patients had adequate organ function, as defined as absolute neutrophil count > 1500/μl, platelets > 75,000/ μl, hemoglobin > 8 g/dl, bilirubin < 2.0 X upper limit of normal, and creatinine ≤ 2 mg% or calculated clearance ≥ 40 ml/mt. To participate in the study patients had to complete at least 4 weeks of chemotherapy. All patients provided consent to participate in the study and the study was approved by the Montefiore Medical Center IRB.

### Administration of chemotherapy, patient evaluation, and follow-up

Patients received oxaliplatin from commercial sources as per standard protocol. For those receiving FOLFOX chemotherapy, oxaliplatin was dosed at 85 mg/m^2^ every two weeks, and for those on XELOX, oxaliplatin was dosed at 130 mg/m^2^ every three weeks. Whole blood (2 tubes with 8 ml each) was drawn at four different time points: 0 hours (pre-dose, prior to oxaliplatin infusion), 2 hours (at the end of the infusion), 48 hours (relative to the beginning of oxaliplatin infusion, at time of pump disconnect, for patients on FOLFOX), and on day 15 (at the beginning of the subsequent cycle, for those patients on FOLFOX). Timepoints of 0, 2, 48 hours and 15 days were chosen for analysis for two reasons: First, we have previously demonstrated that induction of oxaliplatin in cell lines is detected at 24, 48 and 72 hours [26]. Most patients are required to come in after 48 hour (on Day 3) for pump disconnects and hence 48H time point was the most feasible. Also, this study is the first *in vivo* assessment of ERCC1 kinetics and we wanted to observe the changes that occurred in an early (2 hours post-infusion) and delayed fashion (end of chemotherapy cycle- Day 15). Second, since we intended to propose establishing the ERCC1 kinetics as a biomarker, it is important to note the most ideal time point which would give us the information required. Hence a wide range of time points were employed to be able to establish a pattern. Patients who had consented to institutional biorepository for tissue storage were included for SNP analysis (Figure 1).

All patients on chemotherapy underwent a complete medical history evaluation, physical examination, and performance status evaluation within 2 weeks of joining the study, and as per local clinical practice guidelines. Complete blood count with differential test (CBC), a complete chemistry profile, serum tumor markers profile, and urinalysis were performed.

PFS was assessed for patients with mCRC from the time of the first oxaliplatin dose administration to the detection of radiographic or clinical progression. Imaging studies of the chest, abdomen and pelvis were performed after every 12 weeks, and the patient´s response was evaluated using the Response Evaluation Criteria in Solid Tumors (RECIST 1.1) [55]. RFS for surgically resected stages II and III patients was assessed from time of completion of chemotherapy to time of documentation of first relapse or death from any cause.

### Sample collection

Blood was drawn into glass Mononuclear Cell Preparation (CPT) vacutainer tubes (BD Biosciences, Franklin Lakes, NJ), and PBMC were isolated using the Ficoll Hypaque method. The tubes were spun at 1500*g* for 20 minutes to allow the complete separation of the cellular components. Cells were subsequently washed in Phosphate Buffered Saline (PBS) and re-spun. The supernatant was discarded, and cells were washed again in PBS. After a second spin, cells were collected and stored as a pellet at -80˚C.

### RNA isolation and quantitative real-time reverse-transcriptase polymerase chain reaction (qPCR)

RNA was isolated using the RNeasy kit (Qiagen), and 5 µg of total RNA reverse-transcribed using SuperScript III (Invitrogen). *ERCC1* expression was determined by qPCR amplification of 10 ng of cDNA using the SYBR green Core Reagents Kit and a 7900HT real-time PCR instrument (Applied Biosystems), and normalized to the level of glyceraldehyde-3-phosphate dehydrogenase (GAPDH) expression. The primers used in the qPCR assay were as follows: *ERCC1* forward (F): GGAGGCTGTTTGATGTCCTG, and *ERCC1* reverse (R): TTACACTGGGGGTTTCCTTG, *GAPDH* F: TCAAGAAGGTGGTGAAGCAG, and *GAPDH* R: AAAGGTGGAGGAGTGGGTGT. Amplicon sizes of ERCC1 and GAPDH are 80 and 112 bp, respectively.

### Western blotting and quantification of signal intensity

Cells were washed in PBS and lysed at 4º C for 30 min in 200 µL lysis buffer (50 mM Tris-HCL pH 7.5, 150 mM NaCl, 1% NP-40, 0.5% sodium deoxycholate, 1 mM EDTA pH 8), and protease inhibitor cocktail (Sigma catalog number P-8340). The final protein content was determined by spectrometry using a Bio-Rad dye-binding assay (Bio-Rad, Hercules, CA, USA). Samples were boiled for 5 min in Laemmli sample buffer, and 50 µg of protein separated by electrophoresis on 12% SDS–polyacrylamide gels. Proteins were transferred onto the Hybond ECL membranes (GE Healthcare, Amersham Hybond™-P) at 4º C. Rainbow molecular weight markers (GE Healthcare UK Ltd, Cat no. RPN 800E) were used as standards. After the transfer, the membranes were blocked with Tris-buffered saline with Tween 20 (10 mM Tris–HCl, pH 7.4, 150 mM NaCl, and 0.1% Tween 20) containing 10% non-fat milk, for one hour at room temperature, and incubated with anti-human ERCC1 (sc-53281, Santa Cruz Biotechnology, Santa Cruz, CA at 1:200 dilution) overnight at 4º C. The membranes were then washed and incubated with anti-mouse secondary antibody (GE Healthcare UK Ltd, Cat. no NA-931V) in 2.5% blocking buffer, for 1 hour. Membranes were then re-washed, and proteins visualized using ECL Plus Western Blotting Detection System (GE Healthcare UK Ltd, Cat. no. RPN 2132). To control for equal loading, blots were re-probed with an anti-β-actin antibody (Sigma, 1:10,000), for 1 hour at room temperature.

Image quantification was performed using the “Image Studio Lite” software program downloaded from the LI-COR Biosciences website [56]. Increase in optical density of protein band at any time point was considered an increase in expression of the protein in a patient.

### Analysis of SNPs

Genomic DNA was obtained from either the stored cell pellets (from consenting patient samples) or formalin fixed paraffin embedded tissue (FFPE tissue from the biorepository) and was purified using Qiagen DNeasy spin columns. The genomic region harboring the *ERCC1* rs11615 SNP was PCR amplified using Taq PCR Beads (GE Healthcare), using the following primers- *ERCC1* F: GGATCAGGGACTGTCCAGGGTT and *ERCC1* R: CGGGAATTACGTCGCCAAA.

PCR products were then resolved on an agarose gel and gel purified. Gel purified DNA was sequenced by Sanger Sequencing and the genotype of the sample determined by comparison to the following reference sequence: ATCCCGTACTGAAGTTCGTGCGCAA(C/T)GTGCCCTGGGAATTTGGCGACGTAA.

### Statistical analysis

Descriptive statistics were used to summarize patient characteristics. The primary hypothesis tested was that an increase in ERCC1 expression in PBMCs following oxaliplatin treatment will be associated with drug resistance, and a reduced clinical benefit from oxaliplatin in patients with CRC. Analysis for PFS and RFS was carried out by using Graphpad Prism v 8.00 software (GraphPad Software, La Jolla, CA) [57] and reported as Kaplan-Meier curves. The log-rank test was used to compare survival experiences between groups. Categorical variables were analyzed using an exact test. All tests were reported as 2-tailed.
